# Cardiovascular risk and glucocorticoids: a Dutch National Registry of growth hormone treatment in adults with growth hormone deficiency analysis

**DOI:** 10.1007/s11102-024-01448-2

**Published:** 2024-08-31

**Authors:** Tessa N. A. Slagboom, Christa C. van Bunderen, Aart Jan van der Lely, Madeleine L. Drent

**Affiliations:** 1 Department of Endocrinology & Metabolism, Amsterdam UMC location Vrije Universiteit Amsterdam, De Boelelaan 1117, Amsterdam, The Netherlands; 2 Amsterdam Gastroenterology Endocrinology and Metabolism, Amsterdam, The Netherlands; 3 Department of Internal Medicine, Division of Endocrinology, Radboud University Medical Center, Nijmegen, The Netherlands; 4 Division of Endocrinology and Metabolism, Department of Internal Medicine, Erasmus Medical Center, Rotterdam, The Netherlands

**Keywords:** Adrenal insufficiency, Glucocorticosteroids, Hypopituitarism, Growth hormone replacement therapy, Cardiovascular, Cerebrovascular

## Abstract

**Purpose:**

Patients with hypopituitarism are at increased cardiovascular risk, in part because of growth hormone deficiency (GHD), but probably also because of the overuse of glucocorticosteroids in concomitant adrenal insufficiency (AI). We hypothesized that patients with hypopituitarism that were on glucocorticosteroid replacement therapy for concomitant AI would have worse cardiovascular outcomes than those without.

**Methods:**

Retrospective nationwide cohort study. GHD patients from the Dutch National Registry of Growth Hormone Treatment in adults were grouped by the presence (AI; *N* = 1836) or absence (non-AI; *N* = 750) of concomitant AI, and differences between groups were analyzed for baseline characteristics and cardiovascular risk, at baseline and during GHRT.

**Results:**

At baseline, AI patients had higher levels of total and LDL cholesterol (both *p* < 0.01). During GHRT, AI patients were more likely to use cardiovascular drugs (*p* ≤ 0.01), but we did not find worse outcomes for blood pressure, body composition, lipid and glucose metabolism. The risk of developing peripheral arterial disease (HR 2.22 [1.06–4.65]) and non-fatal cerebrovascular events (HR 3.47 [1.60–7.52]) was higher in AI patients, but these differences disappeared in the models adjusted for baseline differences.

**Conclusion:**

We found no clear evidence to support our hypothesis that patients with hypopituitarism and concomitant AI have worse cardiovascular outcomes than non-AI patients. This suggests that glucocorticoid replacement therapy in AI may be safer than previously thought. However, cardiovascular burden, events and medication use at baseline and during GHRT (in unadjusted models) were higher in AI; so the lack of power, the important role of (adjusting for) other risk factors, and the inability to distinguish between glucocorticoid treatment regimens may have influenced the outcomes.

## Introduction

Hypopituitarism is a rare endocrine disorder, in which there is insufficient production of one or more of the pituitary hormones. Hypopituitarism can therefore occur as an isolated pituitary hormone deficiency, but more commonly it occurs in the context of deficiencies in multiple pituitary axes, including the somatotrophic-, corticotrophic-, gonadotrophic-, thyreotrophic axis and arginine vasopressin (AVP) deficiency. In the GENHYPOPIT network, a large international cohort of genetic causes of non-acquired hypopituitarism, multiple hormone deficiencies were present in 74% of the patients [1]. Multiple hormone deficiencies are also common in acquired forms of hypopituitarism, resulting in a heterogeneous group of symptoms and comorbidities [2–4].

One of the most frequently and often earliest pituitary axis to be affected is the somatotroph or growth hormone (GH)/insulin-like growth factor-1 (IGF-1) axis, resulting in growth hormone deficiency (GHD) [1, 2, 5]. Deficiency of the somatotroph axis has adverse effects on body composition (central adiposity, loss of lean body mass), metabolism (worsening of lipoprotein profile) and cardiovascular risk (hypertension, vascular atherosclerosis) [6–8]. Treatment of GHD with growth hormone replacement therapy (GHRT) has a beneficial effect on these manifestations and thus on cardiovascular risk [9].

The prevalence of adrenocorticotrophic hormone (ACTH) deficiency in patients with hypopituitarism varies from 49 to 69%, and ACTH deficiency often presents later than GHD [1–3, 5]. ACTH deficiency leads to adrenal insufficiency (AI), and some of the classic symptoms of AI are opposite to those seen in GHD, such as weight loss, hypotension and hypoglycemia [10]. Patients with AI are treated with glucocorticoid replacement therapy. However, imperfections in treatment regimens with both under- and over-replacement are common, due to the difficulty of mimicking physiological (circadian) cortisol rhythms. Over-replacement of glucocorticoids, resulting in an excess of cortisol, is also detrimental to cardiovascular risk [11, 12] and should therefore be avoided in the treatment of hypopituitarism.

While previous studies have focused on the separate effects of GHRT in GHD and glucocorticoids in AI, it is common in clinical practice to encounter a patient with hypopituitarism suffering from both these deficiencies at the same time. Therefore, the aim of the present study was to determine the concomitant effect of glucocorticoid replacement therapy on cardiovascular outcomes during GHRT in GHD patients from the Dutch National Registry of Growth Hormone Treatment in adults. We hypothesized that patients on glucocorticoid replacement therapy would have worse cardiovascular outcomes than those without, given the common shortcomings of glucocorticoid replacement regimens.

## Materials and methods

### Participants

This retrospective national cohort study included patients from the Dutch National Registry of Growth Hormone Treatment in Adults, whose establishment and patient characteristics have been described in detail previously [13]. Briefly, all patients in the Netherlands with severe GHD who had requested for reimbursement for GHRT from their health insurance since 1998 were included. Data on their characteristics, GHD etiology and treatment, as well as laboratory results, morbidities and concomitant medication use were collected ((bi-)annually) from the medical records by trained monitors until 2009. After approval by an independent committee and obtained informed consent for inclusion of anonymized data in the registry, patients received GHRT from their treating physician.

International consensus guidelines were followed for the diagnosis of severe GHD [14]. The VU University Medical Ethics Committee was consulted prior to the establishment of the registry. However, no subsequent formal judgement was required at that time.

**Fig. 1:**
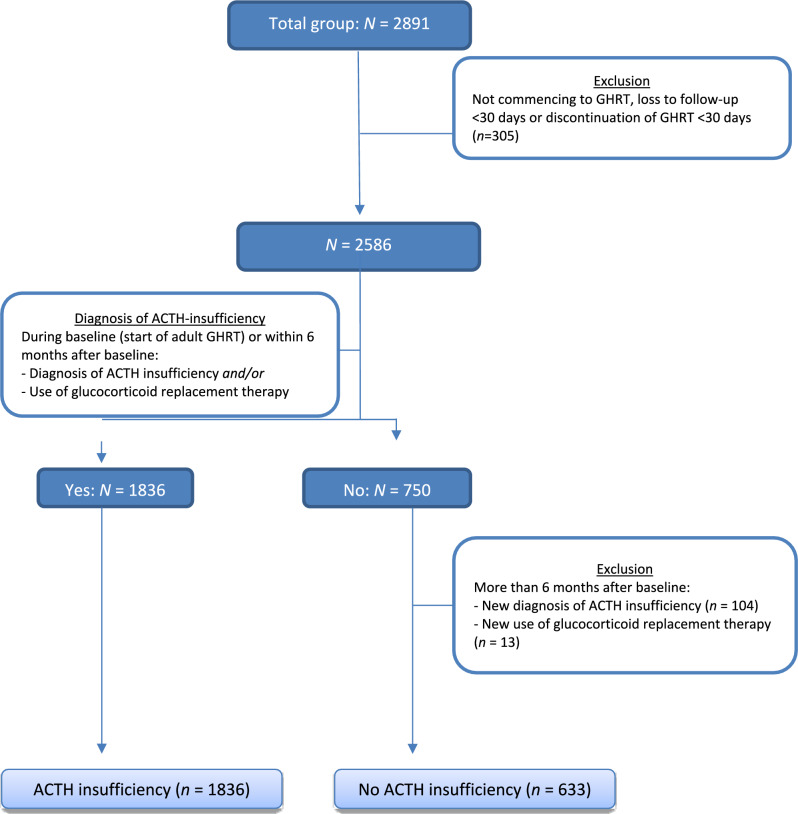
Flow chart of inclusion

The total group consisted of 2891 patients, of whom 305 were excluded because they did not start or discontinued GHRT within 30 days or were lost to follow-up within that period. The remaining group (*N* = 2586) was divided into two subgroups, based on the presence of concomitant AI. Concomitant AI was defined as a diagnosis of ACTH insufficiency and/or use of glucocorticoid replacement therapy at baseline (start of adult GHRT) or within six months of baseline. Of the 2586 patients, 1836 (71%) were diagnosed with concomitant AI. In the remaining group (*N* = 750), 104 and 13 patients were excluded because they received a diagnosis of ACTH insufficiency or were on glucocorticoid replacement therapy after six months from baseline, leaving a group of 633 patients without concomitant AI during follow-up (see Fig. 1). All patients were on GHRT throughout the follow-up period.

### Study outcomes

#### Baseline characteristics

Patient demographics, as well as GHD characteristics, etiology and treatment, other pituitary deficiencies, intoxications, and relevant medical history and medication use at baseline were collected and compared between the groups.

#### Cardiovascular risk profile

Data were collected on the use of cardiovascular (antihypertensive, lipid-lowering drugs, anticoagulant, antiarrhythmic) and diabetes (oral antidiabetic and insulin) medications during follow-up. In addition, patients’ real-life data were collected; blood pressure, body composition (body mass index, waist and hip circumference, waist-hip ratio), lipid profile (total cholesterol, low-density lipoprotein [LDL] cholesterol, high-density lipoprotein [HDL] cholesterol, triglycerides) and glucose metabolism (glycated hemoglobin, HbA1c) at baseline and during annual follow-up. Outcomes for waist and hip circumference, waist-hip ratio, and HDL cholesterol levels were stratified by sex, as men and women have different reference values. Blood pressure and LDL cholesterol outcomes were also stratified according to the use of antihypertensive or lipid-lowering drugs (at baseline or during follow-up).

#### Development of non-fatal cardiovascular events, non-fatal cerebrovascular events, and new-onset diabetes mellitus

The occurrence of non-fatal cardiovascular events, non-fatal cerebrovascular events and new-onset diabetes mellitus was reported by the patient’s physician according to the International Classification of Diseases, 10th Revision (ICD-10). Non-fatal cardiovascular events included coronary artery disease (angina pectoris, coronary sclerosis, myocardial infarction, including cardiac vascular surgery [CABG] and coronary intervention [PTCA]), peripheral arterial disease (intermittent claudication, atherosclerosis, arterial disease, including vascular surgery), arrhythmias (atrial fibrillation of flutter, supraventricular tachycardia, bundle branch blocks or conduction disorder, including cardioversion) and other cardiovascular disease (heart failure, cardiomyopathy, loss of ventricle function, cardiac valve issues, including cardiac valve surgery and other cardiac surgery). Non-fatal cerebrovascular events included cerebrovascular accident (CVA), transient ischemic attack (TIA) and subarachnoid hemorrhage (SAH).

#### Concomitant glucocorticoid medication

Data on the concomitant use of glucocorticoids for indications other than AI, such as inhaled corticosteroids for asthma, topical forms for eczema or systemic forms for rheumatic diseases, were collected and analyzed for their effect on cardiovascular outcomes.

### Statistical analysis

Stata version 15 (StataCorp LLC) and IBM SPSS Statistics version 27 were used for statistical analysis. Numerical data were summarized as mean ± standard deviation (normally distributed data) or median and interquartile range (non-normally distributed data). Comparisons between the AI and non-AI groups were made using *χ*^2^-tests (dichotomous variables), independent *t*-tests (continuous variables with normal distribution) or Mann-Whitney *U*-tests (continuous variables without normal distribution). To assess the effect of GHRT on the course of the cardiovascular risk, linear mixed models (LMM) were used. For the first five years, time was transformed into a one-year categorical variable in the LMM. Subsequently, it was transformed into a five-year categorical variable (10 and 15 years) to ensure a sufficient amount of data. Comparisons between groups (AI versus non-AI) of the effect of GHRT were made by adding interaction terms between time and AI subgroup. To correct for potential confounders, baseline differences in patient demographics, GHD characteristics, etiology, relevant medical history and medication use were added to the LMMs. Cox regression analysis was used to analyze the development of non-fatal cardiovascular events, non-fatal cerebrovascular events and new-onset diabetes mellitus. Kaplan–Meier curves were used to visualize event-free survival. The level of significance was set to *p* ≤ 0.05.

## Results

### Baseline characteristics (Table 1)

**Table 1:** Demographics

			AI (*n* = 1836)	Non-AI (*n* = 633)	*P*
Sex, % female			46%	55%	< 0.001
Age at baseline, years (M ± SD)			44.97 ± 16.35	38.35 ± 16.10	< 0.001
Mean time of GHD, years			5.47 ± 8.13	3.52 ± 6.76	< 0.001
Median duration of AI before start adult GHRT, years			5.17 [13.87–1.58]	N/A	N/A
Etiology GHD	Childhood-onset GHD		19%	33%	< 0.001
	Neoplasm pituitary	Non-functioning adenoma	32%	23%	< 0.001
		ACTH producing (Cushing)	8%	4%	0.004
		GH producing (Acromegaly)	3%	3%	0.975
		Prolactin producing	7%	7%	0.633
	Neoplasm non-pituitary	Benign^1^	18%	5%	< 0.001
		Aggressive^2^	5%	18%	< 0.001
	Iatrogenic^3^		45%	47%	0.377
Pituitary deficiencies	TSH deficiency		91%	39%	< 0.001
	LH/FSH deficiency		86%	45%	< 0.001
	AVP deficiency		26%	7%	< 0.001
	≥ 3 pituitary hormone deficiencies		82%	3%	< 0.001
Previous pituitary treatment	Pituitary surgery		64%	39%	< 0.001
	Pituitary radiotherapy		38%	35%	0.102
Relevant medical history	Cardiovascular risk factors	Hypertension	16%	15%	0.377
		Hypercholesterolemia	11%	8%	0.121
		Diabetes mellitus (1 + 2)	6%	7%	0.785
	Cardiovascular disease	Coronary heart disease^4^	6%	4%	0.042
		Peripheral arterial disease^5^	2%	1%	0.391
		Arrhythmias^6^	2%	2%	0.661
		Other cardiac disease^7^	2%	2%	0.944
	Cerebrovascular disease^8^		5%	3%	0.098
Medication use on baseline	Cardiovascular drugs	Antihypertensives	19%	17%	0.318
		Lipid lowering medication	12%	12%	0.929
		Anti-arrhythmics	0,49%	1%	0.387
		Anticoagulants	9%	6%	0.025
	Glucose lowering medication	Oral	4%	4%	0.624
		Insulin	3%	2%	0.190
Intoxication	Alcohol consumption		24%	28%	0.084
	Smoking	Current	22%	23%	0.564
		Former	15%	9%	< 0.001
Median years of follow-up			5.17 ± 3.96	5.99 ± 4.08	< 0.001
Median dose of received rhGH, mg			0.33 [0.23–0.50]	0.33 [0.21–0.50]	0.645

*AI* adrenal insufficiency, *AVP* arginine vasopressin, *GHD* growth hormone deficiency, *GHRT* growth hormone replacement therapy, *FSH* follicle stimulating hormone, *LH* luteinizing hormone, *non-AI* without adrenal insufficiency, *TSH* thyroid stimulating hormone

^1^craniopharyngeoma, meningioma and cyst (Rathke’s cleft, epidermoid, dermoid, colloid, arachnoid, simple)

^2^astrocytoma, medulloblastoma, (dys)germinoma, chondrosarcoma, leukemia, lymphoma, neuroblastoma, opticus glioma, germ cell tumour, rhabdomyosarcoma, ependymoma, Langerhanscel histiocytosis, (epi/naso)pharynx carcinoma, multiple myeloma

^3^after surgery and/or radiotherapy (pituitary or cranial)

^4^angina pectoris, coronary sclerosis, myocardial infarction, including cardiac vascular surgery (coronary artery bypass grafting, CABG) and coronary intervention (percutaneous transluminal coronary angioplasty, PTCA)

^5^intermittent claudication, atherosclerosis, arterial disease, including vascular surgery

^6^atrial fibrillation of flutter, supraventricular tachycardia, bundle branch blocks or conduction disorder, including cardioversion

^7^heart failure, cardiomyopathy, loss of ventricle function, cardiac valve issues, including cardiac valve surgery and other cardiac surgery

^8^cerebrovascular accident (CVA), transient ischemic attack (TIA) and subarachnoid haemorrhage (SAH)

Compared with non-AI patients, AI patients were more likely to be male, older, have a longer duration of GHD, have more adult-onset GHD and a diagnosis of non-functioning or corticotrophic adenoma, have benign non-pituitary neoplasms and more defects in other pituitary axes, and have had more pituitary surgery. They were more likely to have a history of coronary heart disease, to be taking anticoagulants and to be former smokers. The median dose of rhGH received was the same in AI and non-AI.

### Cardiovascular risk profile at baseline (Table 2)

**Table 2:** Baseline values for cardiovascular risk profile for both groups

			AI	Non-AI	*p*	*p* ^a^
Blood pressure (mmHg)	Systolic	With antiHT	142 ± 21	142 ± 20	0.437	0.145
		Without antiHT	123 ± 16	122 ± 17	0.543	0.197
	Diastolic	With antiHT	85 ± 12	87 ± 11	0.124	0.118
		Without antiHT	77 ± 10	76 ± 10	0.193	0.807
Body composition	BMI (kg/m2)		28 ± 5	28 ± 7	0.932	0.164
	Waist circumference (cm)	Men	101 ± 14	99 ± 14	0.207	0.508
		Women	95 ± 14	94 ± 18	0.665	0.731
	Hip circumference (cm)	Men	103 ± 10	103 ± 11	0.608	0.266
		Women	105 ± 12	104 ± 16	0.781	0.754
	Waist-hip ratio	Men	0.97 ± 0.08	0.96 ± 0.07	0.316	0.610
		Women	0.91 ± 0.09	0.90 ± 0.08	0.369	0.730
Lipid profile (mmol/L)	Total cholesterol		5.76 ± 1.26	5.44 ± 1.14	< 0.001	0.004
	LDL cholesterol	With LLM	3.97 ± 1.36	2.94 ± 1.02	< 0.001	< 0.001
		Without LLM	3.40 ± 0.97	3.38 ± 0.93	0.837	0.706
	HDL cholesterol	Men	1.09 ± 0.37	1.12 ± 0.32	0.447	0.859
		Women	1.40 ± 0.45	1.35 ± 0.39	0.253	0.209
	Triglycerides		2.17 ± 1.66	1.88 ± 1.89	0.018	0.213
Glucose metabolism (%)	HbA1c		5.57 ± 1.05	5.45 ± 0.93	0.154	0.948

*AI* adrenal insufficiency, *non-AI* without adrenal insufficiency, *antiHT* antihypertensives, *BMI* body mass index, *HbA1c* glycated haemoglobin, *HDL* high density lipoprotein, *LDL* low-density lipoprotein, *LLM* lipid lowering medication

^a^ANCOVA with covariates age; sex and growth hormone deficiency onset

At baseline, AI patients had significantly higher levels of total cholesterol and low-density lipoprotein (LDL) cholesterol (in the group of patients using lipid-lowering drugs; *p* = 0.004 and *p* < 0.001, respectively). Triglyceride levels were also higher in the AI subgroup, but this difference lost significance after adjustment for age, sex and GHD onset.

### Cardiovascular risk profile during adult GHRT (Figs. 2, 3 and 4)

**Fig. 2:**
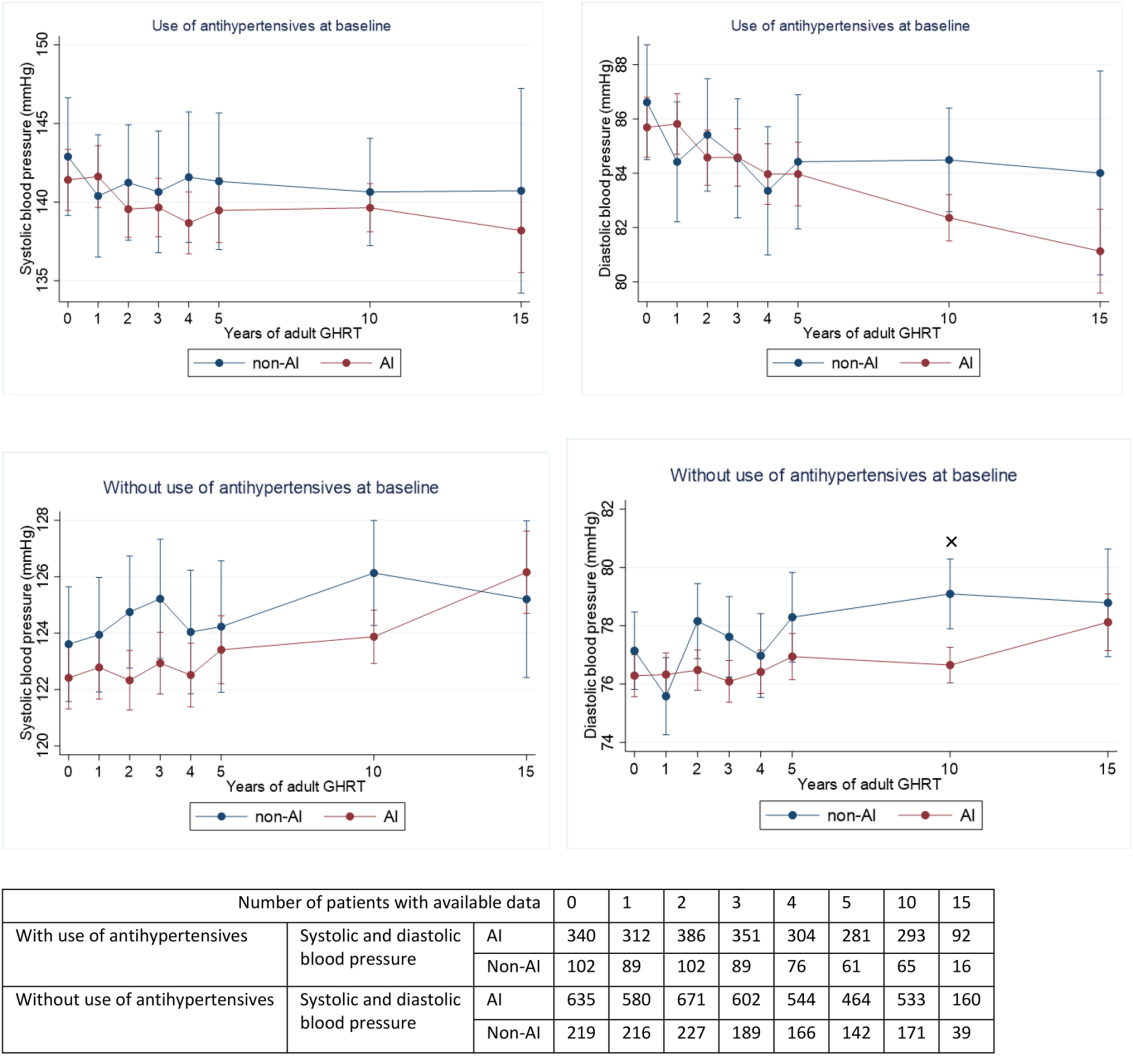
The course of blood pressure during adult GHRT (including 95% confidence intervals). Significant changes between groups (*p* < 0.05) : × = significant only in fully adjusted model (fully adjusted for age, sex, GHD onset, diagnosis of Cushing’s disease, LH/FSH—TSH—or AVP deficiency, history of coronary artery disease at baseline and former smoking)

**Fig. 3:**
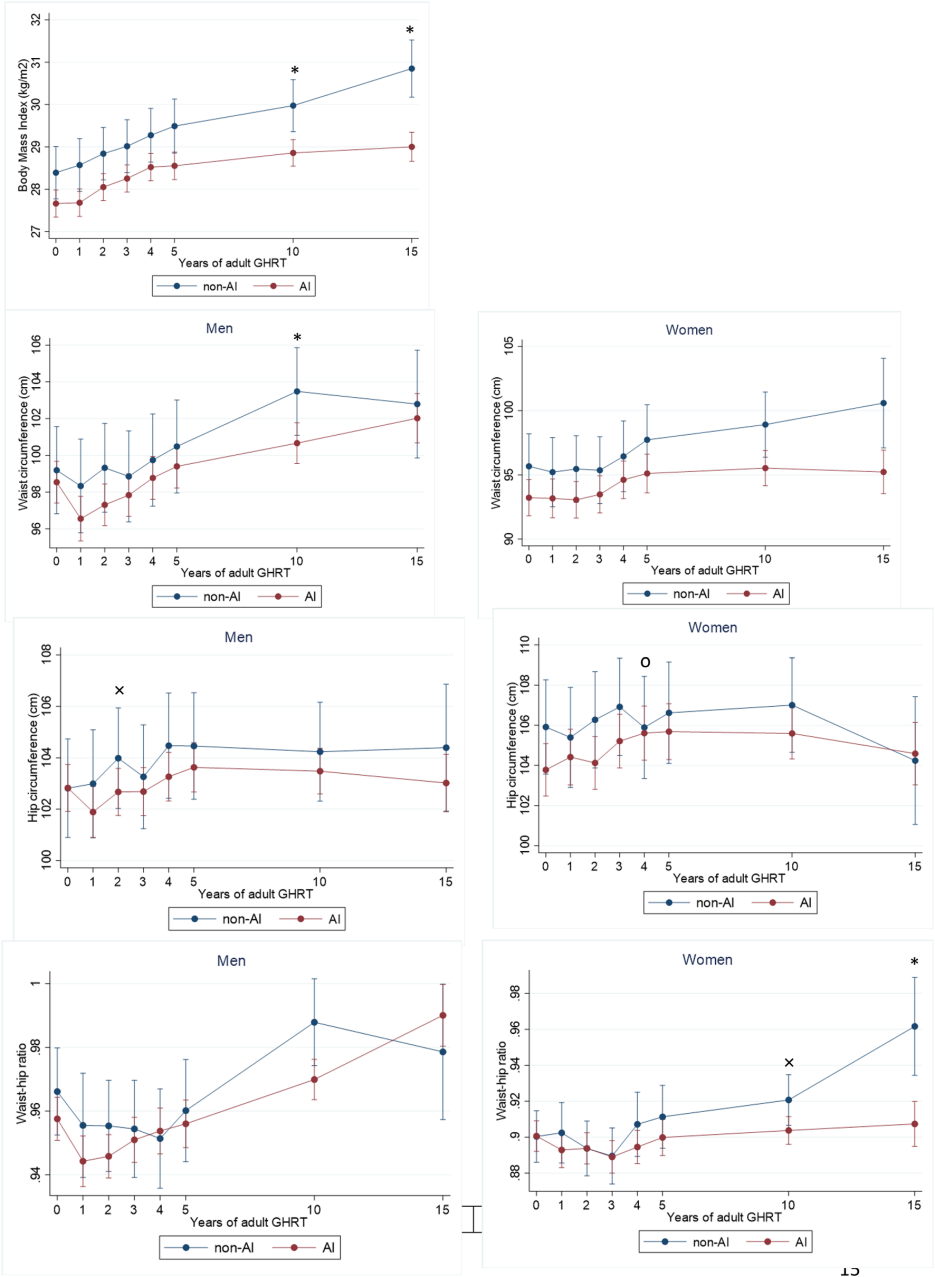

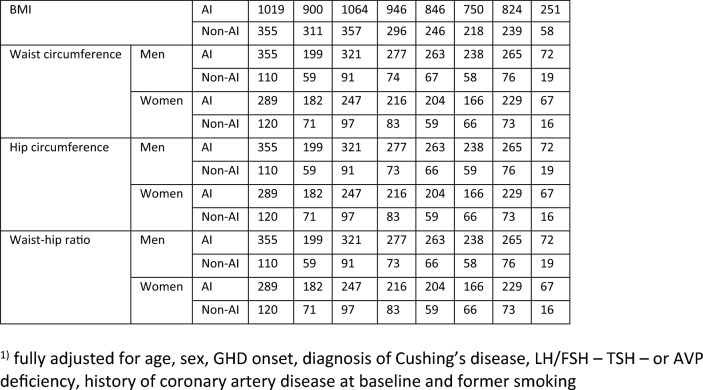
The course of body composition during adult GHRT (including 95% confidence intervals). Significant changes between groups (*p* < 0.05) : о = significant only in unadjusted model; × = significant only in fully adjusted model (fully adjusted for age, sex, GHD onset, diagnosis of Cushing’s disease, LH/FSH—TSH—or AVP deficiency, history of coronary artery disease at baseline and former smoking); * significant in both models

**Fig. 4:**
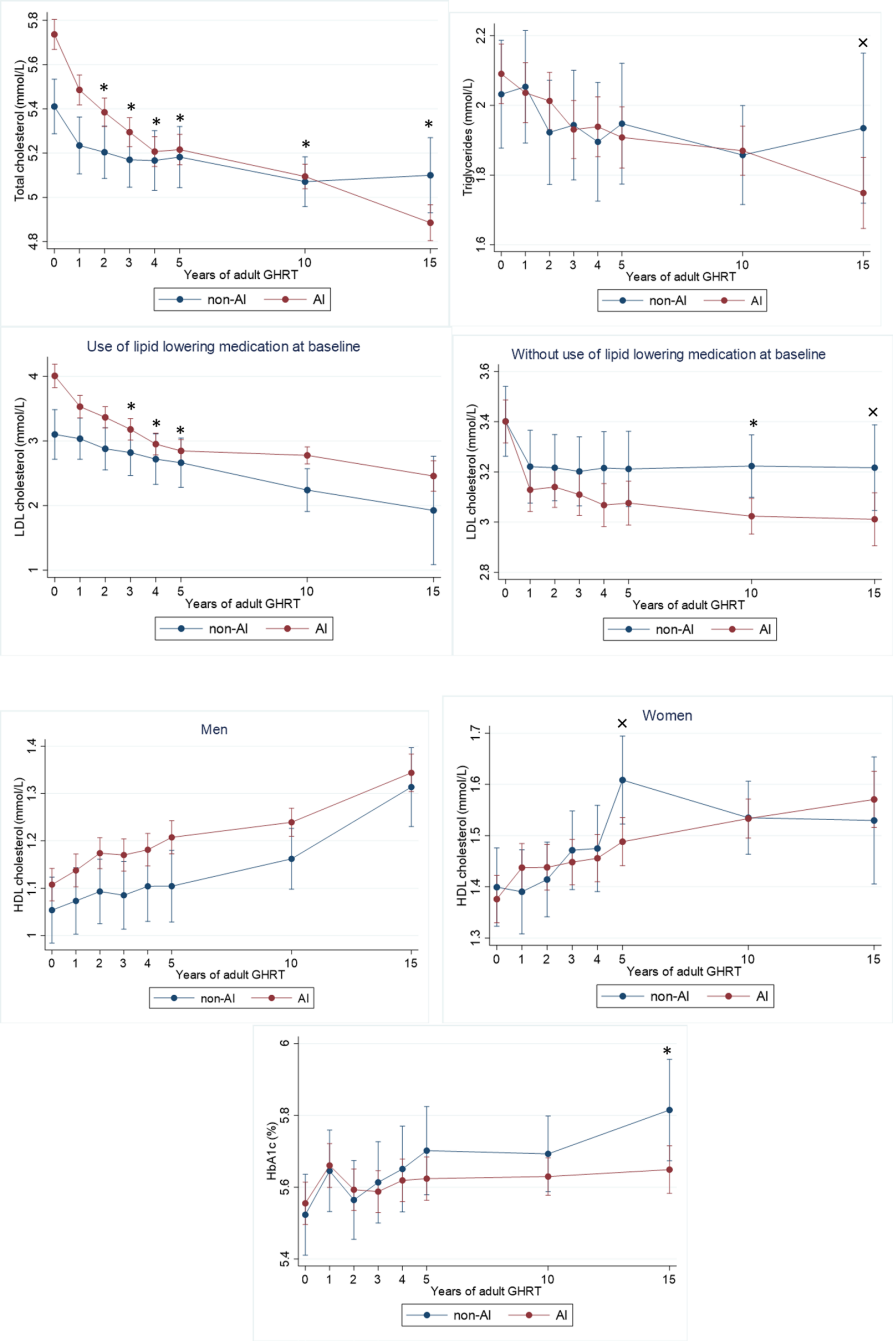

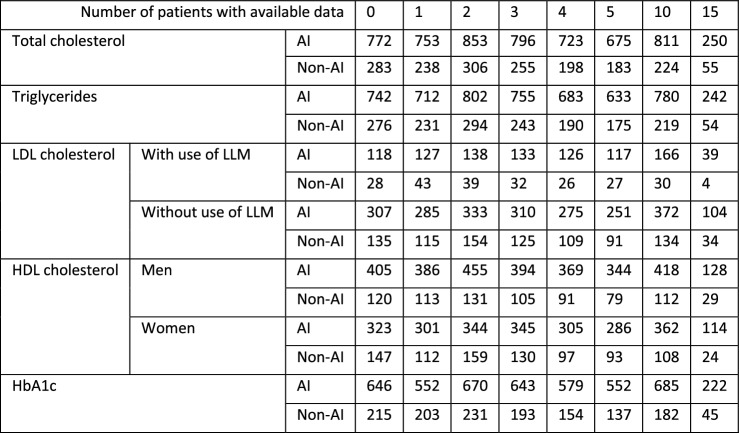
The course of lipid profile and glucose metabolism during adult GHRT (including 95% confidence intervals). Significant changes between groups (*p* < 0.05) : × = significant only in fully adjusted model (fully adjusted for age, sex, GHD onset, diagnosis of Cushing’s disease, LH/FSH—TSH—or AVP deficiency, history of coronary artery disease at baseline and former smoking); * significant in both models

During adult GHRT, AI patients were more likely to use antihypertensives (33% vs. 28%; χ^2^ = 6.37, *p* = 0.01), lipid-lowering drugs (28% vs. 21%; χ^2^ = 11.42, *p* < 0.001) and anticoagulants (18% vs. 10%; χ^2^ = 25.46, *p* < 0.001) than non-AI patients. No significant differences were found in the use of antiarrhythmics or glucose lowering medication (oral diabetics and insulin).

#### Unadjusted models

##### Blood pressure (Fig. 2)

No significant differences between AI and non AI patients were found in systolic or diastolic blood pressure.

##### Body composition (Fig. 3)

Body mass index (BMI): After year 2, BMI increased significantly in both groups and this increase was less in AI patients at years 10 and 15 (*p* = 0.01 and *p* < 0.001).

Waist circumference: Men with concomitant AI had a significantly smaller increase in waist circumference at year 10 (*p* = 0.003) than men without, while no differences were found between the groups in women.

Hip circumference: The increase in hip circumference was greater in women with concomitant AI in year 4 (*p* = 0.05) than in those without concomitant AI.

Waist-to-hip ratio (WHR): In men, no differences in WHR were found between the groups, whereas in women, a significantly smaller increase in WHR was found in AI patients at year15 (*p* = 0.001).

##### Lipid profile and glucose metabolism (Fig. 4)

Total cholesterol: During GHRT total cholesterol decreased in both groups, but this decrease was significantly greater in the AI group from year 2 to year 15 (all *p* < 0.05).

Triglycerides: No significant differences between AI and non AI patients were found.

Low-density lipoprotein (LDL) cholesterol: Results are shown separately for patients with and without using lipid-lowering medication at baseline. Greater reductions in LDL cholesterol were seen in AI patients in years 3 to 5 (all *p* < 0.05) in the group of patients using lipid-lowering drugs, and in year 10 (*p* = 0.01) in the group of patients without lipid-lowering drugs.

High-density lipoprotein (HDL) cholesterol: No differences in HDL were found between the groups in both men and women.

HbA1c: A smaller increase in HbA1c at year 15 was seen in AI patients (*p* = 0.01).

#### Fully adjusted models

The differences compared to the unadjusted models are described below.

##### Blood pressure (Fig. 2)

Among patients using antihypertensive drugs at baseline or during follow-up, the increase in diastolic blood pressure at year 10 was lower in AI patients (*p* = 0.04).

##### Body composition (Fig. 3)

Hip circumference: among women, the difference in year 4 disappeared and no more differences were found in hip circumference between AI and non AI patients, while in men the increase in hip circumference was smaller in patients with concomitant AI in year 2 (*p* = 0.05).

Waist-to-hip ratio (WHR): in addition to a smaller increase in WHR in AI patients at year 15 among women, this was also found at year 10 (*p* = 0.05).

##### Lipid profile and glucose metabolism (Fig. 4)

Triglycerides: The decrease in triglycerides was greater in AI patients at year 15 (*p* = 0.05).

Low-density lipoprotein (LDL) cholesterol: in the group of patients without lipid-lowering drugs, a greater decrease in LDL cholesterol was found in AI patients in year 15 (*p* = 0.05) in addition to year 10.

High density lipoprotein (HDL) cholesterol: in women, a significantly smaller increase in HDL was found in AI patients at year 5 (*p* = 0.05).

### Development of new-onset diabetes mellitus type 2, non-fatal cardiovascular- and cerebrovascular events (Fig. 5; Table 3)

**Fig. 5:**
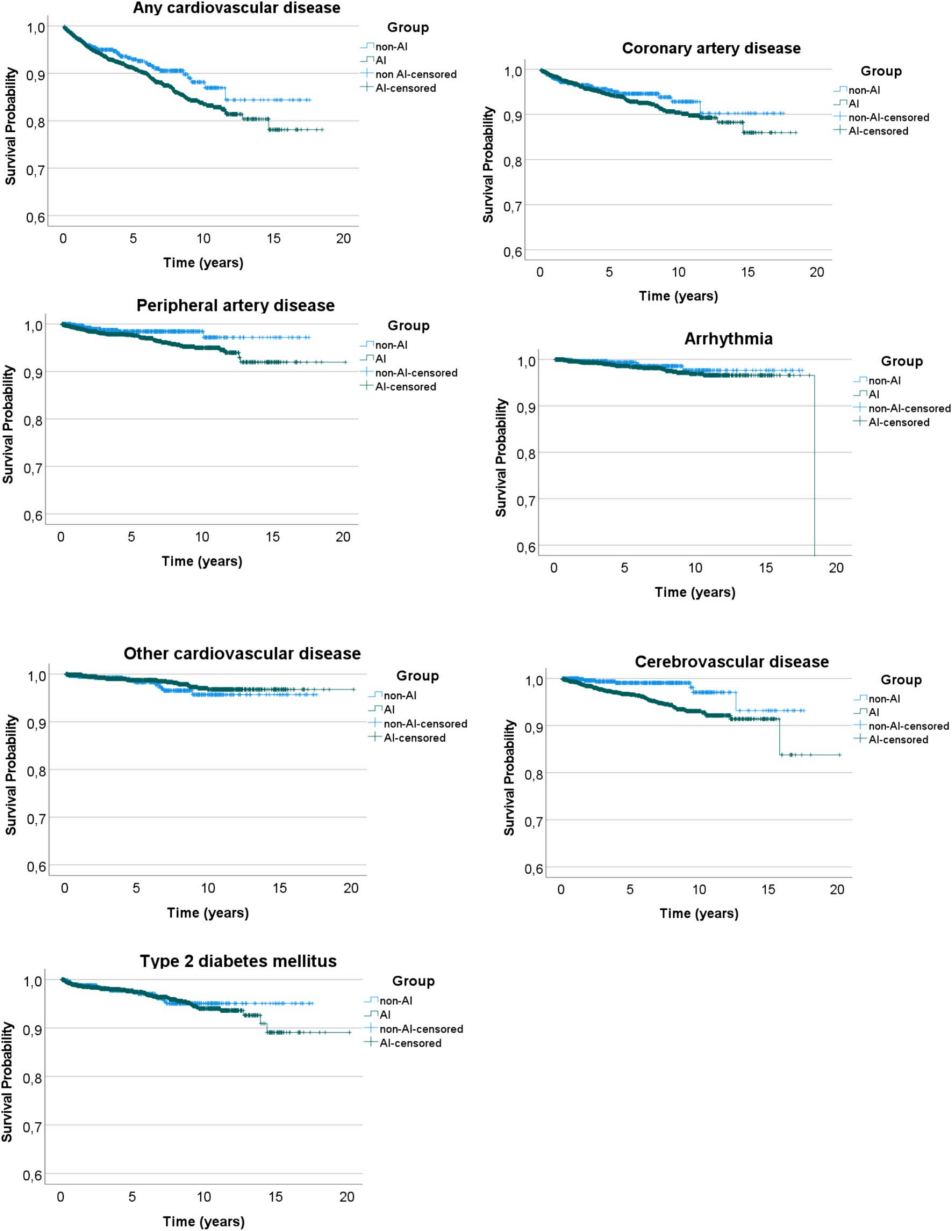
Kaplan–Meier event-free survival curves for non-fatal cardiovascular disease, non-fatal cerebrovascular disease and new-onset type 2 diabetes mellitus

**Table 3:** The risk of developing non-fatal cardiovascular events, non-fatal cerebrovascular events and new-onset type 2 diabetes mellitus according to Cox hazard ratio for both groups

	Number of events		Mean time to event (years)		Unadjusted		Fully adjusted^a^	
	AI (*N =* 1836)	Non-AI (*N* = 633)	AI	Non-AI	HR (95% CI)	*p*	HR (95% CI)	*p*
Cardiovascular, any^1^	189 (10%)	45 (7%)	3.60 ± 3.14	3.10 ± 3.10	1.32 [0.95–1.83]	0.095	0.83 [0.56–1.24]	0.373
Coronary art. disease^2^	112 (6%)	28 (4%)	3.60 ± 3.19	2.65 ± 3.06	1.24 [0.82–1.88]	0.304	0.68 [0.41–1.14]	0.142
Peripheral art.disease^3^	58 (3%)	8 (1%)	4.03 ± 3.51	3.00 ± 3.06	2.22 [1.06–4.65]	0.035	1.25 [0.53–2.95]	0.617
Arrhythmia^4^	32 (2%)	6 (1%)	4.79 ± 3.88	4.46 ± 3.19	1.54 [0.64–3.70]	0.332	1.13 [0.39–3.25]	0.819
Other cardiac disease^5^	29 (2%)	12 (2%)	4.33 ± 3.22	4.33 ± 2.90	0.71 [0.36–1.40]	0.324	0.63 [0.26–1.53]	0.306
Cerebrovascular, any^6^	79 (4%)	7 (1%)	4.17 ± 3.34	5.84 ± 4.59	3.47 [1.60–7.52]	0.002	1.67 [0.57–4.90]	0.348
Diabetes mellitus type 2	63 (3%)	17 (3%)	4.33 ± 3.86	3.19 ± 2.65	1.13 [0.66–1.93]	0.665	0.69 [0.35–1.36]	0.284

*AI *adrenal insufficiency, *art *arterial, *HR *hazard ratio, *non-AI *without adrenal insufficiency

^a^adjusted for: sex, age, onset of growth hormone deficiency (GHD), duration of GHD, diagnosis of non-functioning adenoma/Cushing’s disease/neoplasm non-pituitary benign and aggressive, TSH, LH/FSH and AVP deficiency, former smoking, history of pituitary surgery/coronary heart disease, use of anticoagulants at baseline

^1^coronary heart disease, peripheral arterial disease, arrhythmia and other cardiac disease

^2^angina pectoris, coronary atherosclerosis, myocardial infarction, including cardiac surgery such as coronary artery bypass grafting (CABG) and coronary intervention (percutaneous transluminal coronary angioplasty (PTCA))

^3^intermittent claudication, atherosclerosis, arterial disease, including peripheral vascular surgery

^4^atrial fibrillation or flutter, supraventricular tachycardia, bundle branch blocks or conduction disorder, including cardioversion

^5^heart failure, cardiomyopathy, loss of ventricle function, cardiac valve issues, including cardiac valve surgery and other cardiac surgery

^6^cerebrovascular accident (CVA), transient ischemic attack (TIA) and subarachnoid haemorrhage (SAH)

The Cox hazard ratio’s (HR) showed an increased risk of developing peripheral artery disease (HR: 2.22 [1.06–4.65]) and cerebrovascular disease (HR: 3.47 [1.60–7.52]) in the group of patients with concomitant AI compared with those without (Table 3, Kaplan–Meier curves shown in Fig. 5). However, this increased risk disappeared in the adjusted models. No differences were found in the risk of developing coronary artery disease, arrhythmias, other cardiac disease, any cardiovascular disease, or new-onset diabetes mellitus. There were also no differences in mean time to event (all *p* > 0.05).

### Subanalysis of concomitant glucocorticoid medication (indications other than AI)

#### Total group (AI and non-AI patients)

In the total group, 299 concomitant glucocorticoids were used in 227 (9%) patients during adult GHRT. Of these 227 patients, about a quarter (22%) used ≥ 2 glucocorticoids. Regarding route of administration, 136 patients used inhalers, 38 patients used transcutaneous forms, 35 patients took oral glucocorticoids, 33 intra-nasal and 4 used ocular glucocorticoids. Duration of glucocorticoid use was known for 73 of the 299 medications and had a mean of 696 ± 1163 [2–5717] days.

Cox Hazard ratios showed an increased risk of developing any non-fatal cardiovascular event (coronary heart disease, peripheral arterial disease, arrhythmia and other cardiac disease) in the group of patients using concomitant glucocorticoids compared to patients not using this medication, HR 1.57 [1.10–2.22], *p* = 0.01. No differences were found in the development of the subgroups of non-fatal cardiovascular events (coronary heart disease, peripheral arterial disease, arrhythmia and other cardiac disease), non-fatal cerebrovascular events, or new-onset diabetes mellitus type 2 (all *p* > 0.05, data not shown).

#### Comparisons between subgroups (AI versus non-AI)

There was no statistical difference in the number of patients in the AI (170/1836 = 9%) versus non-AI (57/633 = 9%) subgroup using concomitant glucocorticoids, *χ*^2^ = 0.04, *p* = 0.85.

Within the non-AI group, there were no differences in the development of any non-fatal cardiovascular events, non-fatal cerebrovascular events, or new-onset diabetes mellitus type 2 between patients taking concomitant glucocorticoids and those not taking this medication (all *p* > 0.05, data not shown)

## Discussion

In the current study, we aimed to determine the effect of glucocorticoid replacement therapy on cardiovascular outcomes during GHRT in patients with hypopituitarism. Given the shortcomings of glucocorticoid replacement therapy, we hypothesized that patients treated with glucocorticoids would have worse cardiovascular outcomes than patients not treated with glucocorticoids. In line with this hypothesis, we found that some of the cardiovascular risk factors at baseline were more common in hypopituitary patients with concomitant AI: they were more likely to have a history of coronary artery disease and had higher baseline total - and LDL cholesterol and triglyceride levels. However, contrary to our hypothesis, we did not find clinically relevant worse outcomes in these patients in terms of blood pressure, body composition, lipid and glucose metabolism. The risk of developing peripheral artery disease or cerebrovascular events during GHRT was higher in the AI group, but this difference disappeared in the adjusted models.

Our results are partly consistent with a previous study by Filipsson et al. [15] who included 2424 untreated GHD patients from the KIMS database and evaluated the effect of various glucocorticoid regimens on metabolic outcomes before and after one year of GHRT. Similar to our study, they found that total cholesterol levels were higher in patients on hydrocortisone at baseline (compared to patients not on glucocorticoids) and after one year of GHRT, patients on a hydrocortisone dose of < 20 mg/day did not differ in metabolic endpoints compared to hypopituitary patients without glucocorticoid replacement therapy. In addition, the authors found that patients using cortisone acetate had more favorable metabolic outcomes than those using hydrocortisone or prednisolone/dexamethasone. Moreover, effects on lipid metabolism (total and LDL cholesterol, triglyceride levels) and body composition (BMI) appeared to be dose-dependent in the glucocorticoid group, with an increase at higher doses. A previous open-label, placebo-uncontrolled pilot study tested the hypothesis that the clinical manifestations of hypopituitarism that are currently attributed to GHD (such as altered body composition and worsening of lipid profile) are also the consequence of glucocorticoid over-replacement in these patients [16]. In eleven patients with hypopituitarism and untreated GHD, the daily dose of hydrocortisone was reduced from 20 to 30 mg to 10–15 mg, and after one year there were positive effects on body fat (especially abdominal fat) and lipid profile (reduction in total cholesterol and triglyceride levels), but not on lean body mass and insulin sensitivity. Unfortunately, we were not able to study the dose-dependent or glucocorticoid preparation effect on cardiovascular risk, as this information was not fully available. On the other hand, our results go beyond the previous findings of both mentioned studies because our follow-up time is much longer (15 years versus 1 year). Regarding cardiovascular and cerebrovascular morbidity, we found that, although not significant in the adjusted model, the prevalence of coronary artery disease (6% vs. 4%), peripheral artery disease (3% vs. 1%) or cerebrovascular event (4% vs. 1%) was higher in the group of patients with concomitant AI than in those without. This is in line with previous results from the KIMS database, which found three new cases of myocardial infarction and eight new cases of stroke in the glucocorticoid treated group, compared with no events in the non-glucocorticoid group [15]. In contrast to our study, they also found a higher prevalence of new-onset diabetes in the group of patients on glucocorticoids (*n* = 12) than in those without (*n* = 0). A retrospective cohort study comparing 3948 patients with secondary AI with 67,564 matched controls also showed an increased risk of composite cardiovascular disease (HR 1.10 [1.01–1.19]) and cerebrovascular disease (HR 1.53 [1.34–1.74]), but not for ischemic heart disease (HR 0.91 [0.80–1.04]), after adjustment for comorbidities (type 2 diabetes mellitus, dyslipidemia, previous cardiovascular disease and smoking) [12]. Chifu et al. found that most of the cardiovascular risk factors studied were more common in patients with secondary AI than in those with primary AI, and suggested that this may be related to additional pituitary deficiencies and a higher risk of glucocorticoid over-replacement in the presence of residual adrenal function in secondary AI [17].

Although the introduction of confounders (adjusted models) resulted in some additional beneficial effects on the cardiovascular risk profile in patients with hypopituitarism with concomitant AI receiving glucocorticoid replacement therapy during GHRT, most of these statistical differences are unlikely to be clinically relevant. For example, for most cardiovascular risk factors, a significant difference was found only once at seven time points and represented small changes, such as a difference of 2 mmHg for diastolic blood pressure. Most of the statistical differences found were at years 10 and 15, and the group size was much smaller at these time points, especially in the non-AI group, leading to a larger confidence interval. Contrary to our hypothesis, in the adjusted models we could not show that patients with concomitant AI had worse cardiovascular outcomes than patients without concomitant AI. There could be several reasons for this. The most obvious possible reason is that imperfections in glucocorticoid treatment regimens have less effect on cardiovascular risk in hypopituitary patients than previously thought. One finding that supports this possibility is that previous studies have shown that hydrocortisone availability and cortisol levels are reduced after starting GHRT in hypopituitary patients [18, 19], thereby reducing the risk of glucocorticoid over-replacement. However, multiple studies of glucocorticoid treatment and comorbidities in (active) Cushing’s syndrome, a condition of prolonged cortisol excess, have clearly shown a negative association between cortisol excess and cardiovascular risk [15, 20–22]. Besides, we also found that use of concomitant glucocorticoid medication (i.e. other than glucocorticoid replacement therapy) increased the risk of developing any non-fatal cardiovascular disease. Another reason may be that the population of patients without concomitant AI (*n* = 633) was not large enough to detect possible differences in the risk of developing non-fatal cardiovascular and cerebrovascular events (underpowered). The occurrence of most of these events was more frequent in the AI group, sometimes with quite large hazard ratios, but did not reach statistical significance. As the overall prevalence of these events was low, it may be that a larger population is needed to statistically prove these differences in risk. Furthermore, other confounding factors such as age, primary diagnosis of GHD, extension of hypopituitarism, former smoking and history of coronary heart disease may play a more crucial role in the development of non-fatal cardio- and cerebrovascular events, as the risk of developing peripheral arterial disease and cerebrovascular disease was higher in the AI group on glucocorticoid replacement therapy in the unadjusted models, but lost significance after adjustment for possible confounders. On the contrary, it can also be argued that by adjusting for baseline differences such as history of coronary artery disease and use of anticoagulants, potential effects of AI and its treatment prior to initiation of GHRT are now minimized. Patients with concomitant AI were also more likely to use antihypertensives, lipid-lowering drugs and anticoagulants, all of which reduce cardiovascular risk.

Current AI treatment guidelines recommend a daily dose of 10–25 mg of hydrocortison in adults, with the dose optimized according to patient complaints (symptoms of under- or over-replacement) [23]. Both the total dose and the frequency of administration (i.e. twice or more) therefore vary between patients. Glucocorticoid treatment regimens have also changed over the years. In the past, higher doses (30 mg oral hydrocortisone [24]) were commonly used in the Netherlands, and glucocorticoids other than hydrocortisone (i.e. cortisone acetate or prednisone) were often prescribed. This also applies to the patients in the current study; some of whom may be treated with higher doses and different preparations. It would have been very interesting to analyze the association between glucocorticoid replacement regimens (such as total dose, frequency of administration, different drugs and adherence) and cardiovascular risk, but unfortunately data on these regimens were not fully available in this study. Hypothetically, it could be that only a subgroup of patients receiving glucocorticoid replacement therapy, namely those who are over-replaced, have an increased cardiovascular risk (dose-dependent), and that the undertreated patients, because we could not differentiate in terms of dosage, are now obscuring these results. Another limitation of this study is that there was no method harmonization of laboratory results; blood samples were analyzed in several local laboratories, using various assays with different reference values. This also means that standard deviation scores (SDS) could not be calculated for IGF-1, and therefore we could not compare IGF-1 SD scores between groups and/or include this variable as a confounding factor. However, as GHD was treated and monitored by the patient’s endocrinologist, IGF-1 levels were expected to be within the normal range. The occurrence of non-fatal cardiovascular events, non-fatal cerebrovascular events and new-onset diabetes was based on patient reporting of new medical events to their treating physician. Therefore, some events may have been missed or underreported (such as coronary atherosclerosis), while others may have been misdiagnosed or over-reported (such as angina pectoris).

Overall, we found no clear evidence to support our hypothesis that patients with hypopituitarism who receive glucocorticoid replacement therapy for concomitant AI have worse cardiovascular risk and outcomes than patients without (treated) AI, also treated with GHRT. This may suggest that glucocorticoid replacement therapy in hypopituitarism is safer than previously thought. However, we did find a higher unadjusted risk of developing peripheral artery disease and non-fatal cerebrovascular events during GHRT in the AI group, just as a greater cardiovascular burden at baseline and use of cardiovascular medication during GHRT. Adjustment for the possible effects of AI and its treatment prior to initiation of GHRT (history of cardiovascular disease, use of cardiovascular drugs), as well as, the crucial role of other risk factors (age, sex, primary diagnosis of GHD, deficiency of other pituitary axes, former smoking), the underpowering, and the inability to differentiate between glucocorticoid treatment regimens (daily dose, preparations used, and adherence) may have influenced the results. We therefore recommend that future studies focus on the long-term differences in cardiovascular risk with the various glucocorticoid treatment regimens, which could provide clinicians with valuable information for counselling and management of hypopituitarism.

## Data Availability

No datasets were generated or analysed during the current study.
